# It’s a Small World After All: The Remarkable but Overlooked Diversity of Venomous Organisms, with Candidates Among Plants, Fungi, Protists, Bacteria, and Viruses

**DOI:** 10.3390/toxins17030099

**Published:** 2025-02-20

**Authors:** William K. Hayes, Eric C. K. Gren, David R. Nelsen, Aaron G. Corbit, Allen M. Cooper, Gerad A. Fox, M. Benjamin Streit

**Affiliations:** 1 Department of Earth and Biological Sciences, Loma Linda University, Loma Linda, CA 92350, USA; allenmcooper@gmail.com (A.M.C.); gfox411@gmail.com (G.A.F.); mstreit@students.llu.edu (M.B.S.); 2 Bitterroot College, University of Montana, Hamilton, MT 59840, USA; eric.gren@mso.umt.edu; 3 Biology/Allied Health Department, Southern Adventist University, Collegedale, TN 37315, USA; dnelsen@southern.edu (D.R.N.); acorbit@southern.edu (A.G.C.)

**Keywords:** toxinology, biodiversity, nanomachines, virulence factors, extracellular toxins, exotoxins, predation, chemical defense, chemical competition, definition of venom

## Abstract

Numerous organisms, including animals, plants, fungi, protists, and bacteria, rely on toxins to meet their needs. Biological toxins have been classified into three groups: poisons transferred passively without a delivery mechanism; toxungens delivered to the body surface without an accompanying wound; and venoms conveyed to internal tissues via the creation of a wound. The distinctions highlight the evolutionary pathways by which toxins acquire specialized functions. Heretofore, the term venom has been largely restricted to animals. However, careful consideration reveals a surprising diversity of organisms that deploy toxic secretions via strategies remarkably analogous to those of venomous animals. Numerous plants inject toxins and pathogenic microorganisms into animals through stinging trichomes, thorns, spines, prickles, raphides, and silica needles. Some plants protect themselves via ants as venomous symbionts. Certain fungi deliver toxins via hyphae into infected hosts for nutritional and/or defensive purposes. Fungi can possess penetration structures, sometimes independent of the hyphae, that create a wound to facilitate toxin delivery. Some protists discharge harpoon-like extrusomes (toxicysts and nematocysts) that penetrate their prey and deliver toxins. Many bacteria possess secretion systems or contractile injection systems that can introduce toxins into targets via wounds. Viruses, though not “true” organisms according to many, include a group (the bacteriophages) which can inject nucleic acids and virion proteins into host cells that inflict damage rivaling that of conventional venoms. Collectively, these examples suggest that venom delivery systems—and even toxungen delivery systems, which we briefly address—are much more widespread than previously recognized. Thus, our understanding of venom as an evolutionary novelty has focused on only a small proportion of venomous organisms. With regard to this widespread form of toxin deployment, the words of the Sherman Brothers in Disney’s iconic tune, *It’s a Small World*, could hardly be more apt: “There’s so much that we share, that it’s time we’re aware, it’s a small world after all”.

## 1. Introduction

Toxinologists and other biologists have studied toxic organisms and their secretions for centuries [1,2,3,4]. Their interest has stemmed in large part from the frequently severe consequences resulting from human exposure [5,6]. Humans have also tapped the potential of toxins to explore treatments for human ailments and diseases [7,8,9]. In doing so, they have leveraged countless natural experiments involving interactions of the toxins with targeted cells and tissues.

The classification of biological toxins, in particular the distinction between poison and venom, features a colorful and sometimes contentious history (reviewed by Nelsen et al. [10], but see also more recent views [11,12,13,14,15,16,17] and our Conclusions section). Nevertheless, with a consensus opinion and the introduction of a third term [10], toxic biological secretions can be classified into three groups based on the mode of delivery to other organisms. These include poisons transferred passively without a delivery mechanism (e.g., ingestion, inhalation, or absorption across the surface); toxungens delivered to the body surface without an accompanying wound (e.g., spitting, spraying, or smearing); and venoms conveyed to internal tissues via the creation of a wound (e.g., stinging, biting). Organisms that possess these toxins are referred to as poisonous, toxungenous, and/or venomous, respectively. These distinctions provide a meaningful framework for studying the evolution of these toxins, including their biochemical structure; associated structures for their synthesis, storage, and application; and their functional roles.

Discourse regarding venoms and venomous animals has focused almost exclusively on animals. Venom usage has evolved independently in at least 104 lineages among at least eight animal phyla, which highlights the remarkable adaptive utility of the trait [18]. But do venom delivery systems exist in other entities? Although certain plant groups are occasionally referred to as venomous, they are generally not recognized as such, and the same can be said for other major groups, including fungi, protists, bacteria, and viruses. We briefly argued earlier that venomous representatives likely exist among each of the aforementioned groups, but we cited only a few examples [10].

Here, we undertake an expanded exploration of the diversity of venom delivery systems among various biological entities. We begin by describing key attributes of venoms and venom delivery systems in animals, and then conduct a search for analogous systems among other entities, namely plants, fungi, protists, bacteria, and viruses. If fully analogous systems exist in groups other than animals, we should apply the same terminology without hesitation [10]. Should we find that many lineages other than animals have similarly invented the use of venom, we might better appreciate just how remarkable an innovation it is.

## 2. Key Attributes of Venoms, Venom Delivery Systems, and Venomous Organisms

Venomous animals all share one critical feature in common: they introduce a toxic secretion into the internal milieu of another organism through a wound generated by a fang, spine, or other such structure [10]. However, the acquisition, storage, delivery, and function of the secretion varies remarkably among different animal groups.

Many venomous animals synthesize and store toxic secretions within their own cells and/or tissues. Autoaglandular–venomous animals synthesize their own toxins but lack glands to store them [10]. These are often relatively simple animals, like cnidarians (e.g., anemones, corals, jellyfishes) that possess specialized stinging cells (cnidocyte) containing both the toxic secretion and the delivery system (a harpoon-like stylet). Autoglandular–venomous representatives are generally more complex and usually synthesize and store their secretion within one or several glands [10]. Scorpions, for example, possess two glands and expulse the contents through ducts to the stinger (aculeus) that penetrates the target.

Some venomous animals, however, expropriate and store the toxic secretions of others. Heteroaglandular–venomous animals acquire toxins from other organisms and lack storage within glands [10]. Certain crabs, for example, co-opt the nematocysts of anemones by situating the entire anemone on their carapace or within their claws. Heteroglandular–venomous animals store their exogenously acquired toxins within glands [10]. A number of marine worms, for example, sequester tetrodotoxin produced by symbiotic bacteria within their glands and deliver it through a wound for predation and possibly defense. In many cases, species that possess exogenously acquired toxins can be facultatively venomous, with some individuals possessing the toxins, sometimes temporarily, and others lacking them.

Venoms often comprise multiple toxins that act individually and/or synergistically to target essential components of normal physiological and signaling processes. Although their function relates largely to predation and defense, venoms can serve other purposes, including mating, offspring care, intraspecific communication, intraspecific competition, habitat creation, antimicrobial ointment, and ectoparasite deterrence [4,18]. Because the secretion comprises a limited commodity, numerous animal groups judiciously deploy their venom, a strategy known as venom metering and sometimes venom optimization [19,20,21,22].

Collectively, these attributes of venom and venom delivery systems offer meaningful comparisons when seeking analogous systems in non-animal groups.

## 3. Plants

**Figure 1:**
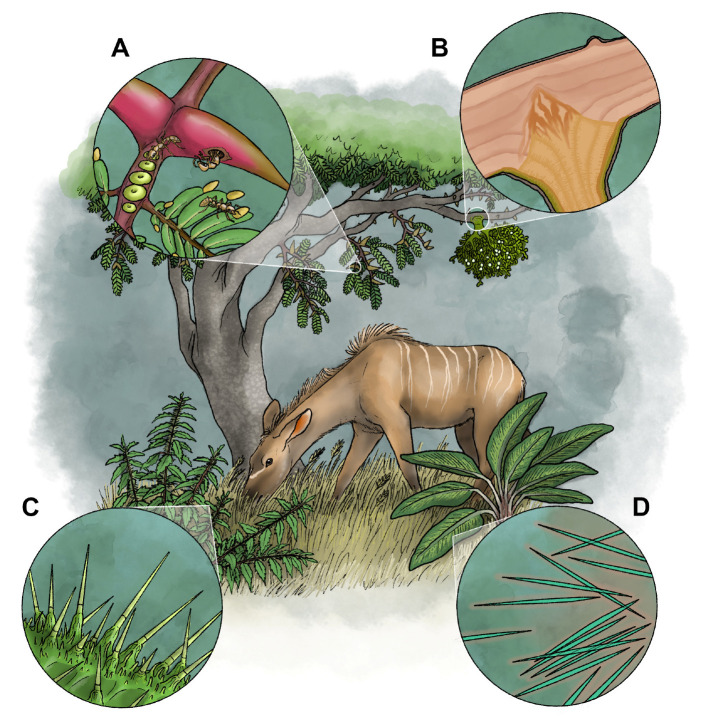
Four representative plant species showcasing proposed venom delivery systems. (**A**) *Acacia* (*Vachellia*) *cornigera*. Inset shows specializations for hosting colonies of symbiotic ants, including domatia (living quarters for the ants), extrafloral nectaries (nectar-producing glands), and Beltian bodies (providing food rich in lipids, sugars, and proteins and often red in color). The ants are venomous and protect the plant from herbivores, providing an effective defense analogous to that of facultatively venomous animals which co-opt the toxins of others. (**B**) *Viscum album*. Inset shows a cross-section of the specialized haustorium root structure invading the host plant’s vascular cambium. The haustorium secretes enzymes that degrade the protective bark layer and stimulate growth of new xylem tissue to connect with the parasite’s own vasculature. (**C**) *Urtica dioica*. Inset shows stinging trichomes, which comprise hollow, hypodermic needle-like structures which penetrate and break off in an animal’s skin upon physical contact, releasing irritating toxins. (**D**) *Dieffenbachia* sp. Inset portrays specialized calcium oxalate crystals (raphides) which penetrate the mucous membranes of animals that feed on the plant, causing irritation and potentially introducing proteolytic enzymes or pathogenic bacteria and fungi. Artwork: M. Benjamin Streit.

Plants comprise diverse terrestrial forms in the more exclusive concepts of delineation and are largely autotrophic. As such, they do not normally capture prey (there are exceptions), and therefore are more likely to employ toxins for defense rather than predation. Plants rely on structural protection as the first line of defense against herbivory, either through the formation of a waxy cuticle and/or the development of trichomes, spines, and setae [23]. Pubescence consists of the layer of hairs (trichomes) extending from the epidermis of the above-ground plant parts, whereas spinescence includes structures such as spines, thorns, and prickles [24]. As a second line of defense, many plants also produce secondary metabolites [23,25,26]. These chemicals are toxic upon ingestion [27], and plants that possess them are typically considered poisonous rather than venomous. However, one group of pubescent plants dispenses their toxins through puncture structures. A second group of spinescent plants possesses puncture structures but delivers pathogenic bacteria and fungi rather than toxins to defend itself. A third group seeks protection by enticing venomous symbionts (ants) to do their bidding. The latter two examples of exogenous-sourced toxin deployment are functionally similar to many animals that protect themselves with toxins acquired from other organisms. Each of these three mechanisms of defense could be considered candidate venom delivery systems, and we will describe each in turn (Figure 1). A fairly sizeable group of parasitic plants, some of which are fully heterotrophic, inserts special organs into other plants or fungi to extract water and nutrients. We also discuss this nutritional mode as a possible venom delivery system.

### 3.1. Stinging Trichomes

Plants from at least five families representing some 650 species [28] are equipped with sharply pointed hair-like structures called stinging hairs or stinging trichomes to protect against arthropod, reptilian, mammalian, and possibly avian herbivores (Figure 1C). Other trichomes exist as irritant hairs that cause mechanical damage only. Two major types of stinging trichomes exist: *Urtica*-type stinging hairs, with the classical “hypodermic syringe” mechanism expelling only liquid, and *Tragia*-type stinging hairs, expelling a liquid together with a sharp crystal [28]. These plants usually possess additional mechanical and/or chemical protection [28].

When an herbivore brushes against one of these plants, the hollow, hypodermic needle-like, mineralized (except in one genus) end of the hairs penetrate and break off in the animal’s skin, releasing toxins. Immediate pain appears to result, at least in part, from the variable presence of three neurotransmitters: acetylcholine, histamine, and serotonin [29]. Inorganic potassium salts may abound in the fluid and may further induce or synergize the pain [28]. These effectors, and likely others that are yet unknown, quickly unleash itching, tingling, burning, piloerection, arterial dilation, local sweating, rash development, and even neuropathy [29,30,31,32]. The intense pain generally peaks after 20–30 min, but some symptoms can persist for days or even months [30,32]. Stinging trees of the genus *Dendrocnide*, widely considered the most draconian within the group, possess neurotoxins (gympietides [33]) and other effectors that have been implicated in the deaths of livestock, dogs, and at least one human [30,31,33,34,35,36]. Stinging hairs are apparently ineffective against invertebrate pests [28].

If the pedestal cells at the base of the stinging cell can be considered a gland [37,38], these plants would be autoglandular–venomous, with delivery and a functional role of venom identical to that of many sessile animals (e.g., cnidarians, bryozoans, crinoid echinoderms). Somewhat analogous to venom metering by animals, these plants increase the density of stinging hairs in response to herbivore browsing [39], which comprises a form of inducible defense [40].

### 3.2. Puncture Structures (Thorns, Sines, Prickles, Silica Needles, and Raphides)

Many plants also inject pathogenic bacteria and fungi into herbivores via thorns, spines, and prickles (Figure 1D) [41,42]. These puncture structures are frequently aposematic (conspicuously colored), advertising their anti-herbivorous function [43]. They can also harbor diverse pathogenic bacteria and fungi that may preferentially reside on the structure [41,42]. In addition to these larger structures, a vast number of plants produce within their tissues sharp-pointed microscopic structures known as silica needles (a type of phytolith) and raphides (calcium oxalate or calcium carbonate crystals). These structures can potentially introduce pathogenic bacteria, fungi, and/or toxins into herbivores via injury of their sensitive mouth lining and digestive tracts [44,45,46]. While the larger puncture structures and probably the toxins incite immediate pain, the pathophysiology caused by introduced microorganisms requires more time to develop. Nevertheless, some animal toxins, including venoms, do not exert immediate effects in certain contexts (e.g., defensive bites to mammals by certain snakes [47]) and the time frame for symptoms in target animals has not been incorporated into formal definitions of venom [27]. Halpern et al. [42] proffered the possibility that bacteria secrete toxins on the surface of puncture structures.

### 3.3. Venomous Symbionts (Ants)

Another large group, comprising at least 681 species (probably more than 1100 based on modeling) in 159 genera and 50 families [48], coexists with colonies of stinging ants that provide effective, venomous defense of the plant itself (Figure 1A) [49,50]. Known as myrmecophytes, these plants provide living quarters (called domatia) and produce food rewards (e.g., extrafloral nectaries, fruit pulp, and/or Beltian bodies) to attract and sustain the ants, often in an obligate mutualism [49,50,51,52]. Domatia-bearing plants occur almost exclusively among angiosperms from tropical regions, but include one family of ferns and no gymnosperms [48]. Ancestral reconstruction implies 158 independent origins and 43 losses of domatia [48], which exceeds the conservative estimate of 104 independent origins of venom identified in animals [18].

Ant-plants are unambiguously heteroaglandular–venomous organisms, with toxin delivery provided by their symbiotic ants that are sometimes referred to as bodyguard ants. They are fully analogous to other heteroaglandular–venomous animals, such as crabs that co-opt the toxins of anemones for self-protection. Somewhat analogous to venom metering by animals, ant-plants can increase the size of their ant colonies in response to herbivory by producing additional food and can by similar means manipulate ants to preferentially frequent the younger leaves [53,54,55].

### 3.4. Haustoria of Parasitic Plants

Some 4100 species among at least 15 families of flowering plants parasitize other plants, extracting at least some of their water and nutrition from their victims [56]. Another 400 or so species parasitize mycorrhizal fungi and are known as myco-heterotrophs rather than parasitic plants [57,58,59]. Many plant-plant parasites tap into the conducting system of host plants via penetrating root organs called haustoria (singular is haustorium; Figure 1B). Some plant parasites possess chlorophyll and are capable of photosynthesis (hemiparasitic), whereas others are fully dependent on their host (holoparasitic [60,61]). Although some parasites reside in the host, many remain on the surface, linked by the haustoria that vary in structure depending on lifestyle [62]. As penetrating structures that create a wound in the host plant, the haustoria can serve as a conduit for toxin delivery.

Parasitic plants and their hosts presumably engage in complex competitive chemical interactions. Germinating parasites seek hosts via the detection of volatile chemicals [63] that can involve even neighboring potential hosts. The mechanism of host parasitism has been characterized for the genus *Cuscuta* [64]. These plants are sensitive to terpenoids produced by tomato plants and use them to locate a potential host. After a suitable host has been found and initial contact made, the plant develops prehaustoria, also known as adhesive disks, that secrete adhesive substances such as pectins and other polysaccharides to reinforce the adhesion. The cells of the host plant respond with an increase in cytosolic calcium that lasts 48 h; however, the exact function of this response remains unknown. In addition, the host plant also produces its own sticky substances, such as arabinogalactan proteins, which further adhesion. The haustoria then penetrates the host tissues, which involves the release of hydrolytic enzymes such as methylesterases and complexes of lytic enzymes consisting of pectinases and cellulases that degrade the host cell walls. Once inside the host, the haustoria form “searching hyphae”, which try to reach the phloem and xylem of the host. Once the vascular tissues are reached, chimeric cell walls composed of the host and parasite are formed, and interspecific plasmodesmata build up a cytoplasmic syncytium between *Cuscuta* and the host plant.

Because *Cuscuta* releases a toxic secretion through a wound to damage another plant, it can be regarded as an autoaglandular–venomous organism. While *Cuscuta* provides one example of how a host–parasite connection is formed, much remains to be learned about the mechanisms of host invasion among other parasitic plants, including the chemicals used to influence their host’s behavior and avoid retaliation.

## 4. Fungi

**Figure 2:**
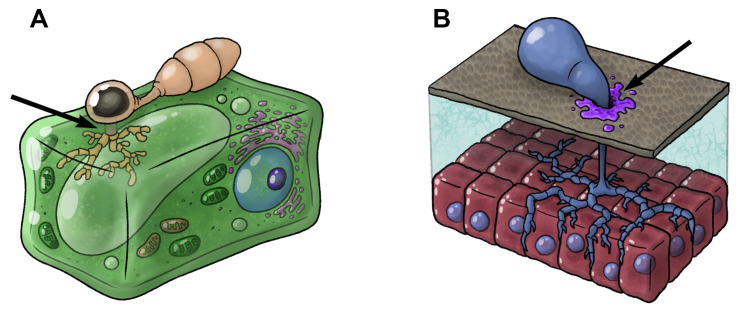
Two proposed venom delivery systems in fungi. (**A**) The appressorium of phytopathic taxa produces a peg-shaped structure (indicated by arrow) which penetrates the plant’s cell wall, allowing the fungal hyphae to deliver toxins into the target plant. (**B**) Entomopathic fungi use appressoria, adhesives, and/or cuticle-degrading enzymes (indicated by arrow) to create a wound through which the fungal hyphae can enter the tissues of the host and deliver toxins. Artwork: M. Benjamin Streit.

Fungi comprise a large group of eukaryotes that includes both single-celled species and more familiar multicellular organisms such as mushrooms. Fungi are widely distributed, having colonized virtually every habitat, including the ocean [65,66]. Most fungi are inconspicuous due to their diminutive size and cryptic lifestyles in soil, on dead matter, or in frequent symbiosis with plants, animals, or other fungi. Some fungi are parasites that cause pathogenesis [67,68], and others are predators of nematodes [69,70]. To support these modes of nutrition, many fungi produce toxins that fall into an assortment of different classes [71]. Many such toxins comprise nonvolatile secondary metabolites (often called mycotoxins) that are toxic upon ingestion by animals [71], but some fungi deliver these or other toxins via hyphae into infected hosts for nutritional and/or defensive purposes. Specialized penetration structures create a wound to facilitate toxin delivery. Thus, the combination of a wound and toxin delivery (sometimes independent of hyphae) comprises a not-so-crude and previously unappreciated venom delivery system [10]. We describe and illustrate these systems in two groups of relatively well-studied fungi (Figure 2).

### 4.1. Phytopathogenic Fungi

This group enters plants by using appressoria, which are specialized non-hyphal cells that form a minute peg and penetrate the cuticle of the plant via turgor pressure (Figure 2A) [72]. As the hyphae penetrate, they secrete toxins that destroy the plant’s cells to derive nutrition from the dead cells and to protect against the host’s defense [72]. In at least one case, an endosymbiotic bacterium produces the toxin, and the metabolically lean host fungus cannot reproduce without the bacterium [73]. This latter example is analogous to that of many animals which utilize toxins synthesized by bacteria as venom.

### 4.2. Entomopathogenic Fungi

This group uses appressoria, adhesives, and/or cuticle-degrading enzymes to penetrate the cuticle of insects [74,75]. Among the toxins delivered by penetrating hyphae, one group, the destruxins, clearly damages the host. The penetrating hyphae deliver at least six groups of toxins that act by directly inhibiting critical cellular activities (e.g., oxidative phosphorylation and ATPase activity) and by impairing the insect’s immune system, rendering the insect host vulnerable to bacteria [75,76,77,78].

### 4.3. Nematophagous Fungi

Certain individuals within this group possess an astonishing diversity of capture and penetration devices for preying upon vermiform nematodes. These devices include specialized hyphae that form adhesive networks, adhesive knobs, columns, sticky balls, and constricting and non-constricting rings that adhere to, and can even lasso, their mobile prey [79,80,81]. Many of these nematophagous fungi produce toxins to paralyze or kill the nematodes in either or both predatory or defensive contexts, with the toxins acting topically or penetrating the pierced cuticle of the victim [69,79,80,81,82,83,84,85].

Representatives among these three groups clearly illustrate that fungi employ toxins as autoaglandular–venomous and sometimes heteroaglandular–venomous organisms. Recently, humans have manipulated fungal genes to facilitate delivery of neurotoxins isolated from scorpions into target organisms, such as insects, for pest control [86]. These biopesticides comprise novel venom delivery systems that may be much safer environmentally than chemical pesticides. In this unusual application, both the fungus (bioengineered) and the human (facultatively) may be regarded as autoaglandular–venomous organisms [10].

## 5. Protists

**Figure 3:**
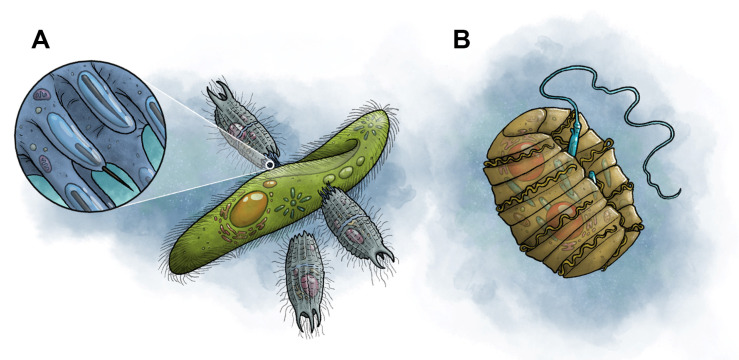
Two proposed venom delivery systems in unicellular eukaryotes. Both are offensive extrusomes that discharge their contents outside of the cell. (**A**) A group of ciliates (*Coleps*) attacking a *Paramecium* using venom. Inset shows the toxicysts, specialized organelles that penetrate the cell membrane of the target and deliver toxins. (**B**) The dinoflagellate *Polykrikos* displaying a discharged nematocyst, a harpoon-like organelle that potentially delivers venom into target prey and structurally resembles the nematocysts of venomous animals in the phylum Cnidaria (e.g., anemones, corals, jellyfishes). Artwork: M. Benjamin Streit.

Protists comprise a diverse group of eukaryotic microorganisms that are mostly unicellular. The multicellular forms lack specialized tissues, which separates them from the other eukaryotes. Protists thrive in nearly every environment that includes liquid water, where they exhibit a broad range of cellular structures, locomotion modes, and lifestyles. Like bacteria, they also occupy a range of different trophic levels. Many of the predatory forms obtain nutrition using mechanisms that bear surprising resemblance to those of venomous animals. We summarize two examples that represent candidate venom delivery systems among autoaglandular–venomous protists (Figure 3).

### 5.1. Extrusosomes

Extrusomes are membrane-bound bodies usually present in the cell cortex that occur widely among protists. As extrusive (ejectable) bodies, they discharge their contents outside of the cell in response to mechanical or chemical stimuli while leaving their host cell intact. Although astoundingly diverse in form and function, extrusomes generally serve defensive and/or predatory functions. One class of offensive extrusomes, the toxisomes, functions in a predatory context to capture and kill prey through the involvement of toxins [87]. They are generally positioned near the oral apparatus, where they can make initial contact with the prey during raptorial feeding and discharge within milliseconds. These toxin-filled, harpoon-like projectiles rupture the victim’s cell membrane and expel toxins (Figure 3A), resulting in paralysis or death of the target [87,88,89]. Composition of the toxins remains poorly understood due to the challenge of acquiring and purifying them, but acid phosphatase has been identified in some raptorial ciliates, which is present in the lysosomes of animal cells and may initiate the digestive process [90,91]. These actions bear a striking resemblance to those of the cnidarian nematocyst despite the substantial difference in size. They also differ from the activity of other projectile structures, such as trichocysts used by *Paramecium* and other ciliates, in which the ejected structures trail non-toxic, filamentous proteins to ensnare rather than envenomate their prey.

Nematocysts comprise another class of offensive extrusomes (Figure 3B). Morphologically and functionally very similar to the nematocysts possessed by the cnidarian genus *Hydra*, the nematocysts of *Polykrikos* fire based on mechanical stimulation, and discharge rapidly, resulting in eversion of a stylet and thread [92,93]. Nematocyst penetration of prey is associated with rapid cessation of prey movement [92,93,94,95]. At present, no toxins are known to be associated with the dinoflagellate nematocyst, as it has only been observed to function as a harpoon [93]. However, based on functional and morphological similarities to animal nematocysts, toxins may well be present.

### 5.2. Karlodinium Karlotoxins

Roughly half of the free-living dinoflagellates exhibit mixotrophy, relying on a mixed nutritional mode of both photosynthesis and phagotrophy [96]. To obtain food via phagotrophy, several species of the genus *Karlodinium* possess a suite of toxic compounds, known as karlotoxins, which exhibit hemolytic, ichthyotoxic, and cytotoxic properties [97]. These toxins stun their prey, which are primarily microalgae, but can also include metazoans [96]. Although the mechanisms by which the toxins act remain largely unknown, some function by generating pores in target cell membranes containing desmethyl sterols, causing increased ionic permeability resulting in membrane depolarization, disruption of motor functions, osmotic swelling, and lysis [98,99]. This mode of killing comprises toxungen use, with application of the toxin to the target’s external surface. However, Berge et al. [96] suggested toxins might also be transferred via extrusosomes covering *Karlodinium*’s cell surface or through the peduncle (feeding tube) that pierces the prey to facilitate ingestion. An experimental study demonstrated that *K. australe* more effectively kills rotifers when in contact with their prey compared to the presence of toxins only in the water, suggesting that the toxins can be delivered to internal tissues [100].

Other dinoflagellates exhibit similar carnivorous behavior, but it remains unexplored whether the toxins act as toxungens via surface contact [101] or as venoms via the creation of a wound in the prey. Dinoflagellate toxins also confer a protective (anti-grazing) function against microzooplankton and copepod predators [102,103], though the mechanisms of toxin delivery need further study.

## 6. Bacteria

Bacteria exhibit a diverse range of behaviors that we generally associate only with animals. These behaviors include surprisingly complex forms of predation (e.g., wolf-pack hunting and cannibalism), antipredatory behavior, migration, and social behaviors such as communication, competition, cooperation, heating, altruism, and spite [104,105,106,107,108]. Chemicals, including toxins in some cases, generally mediate these behaviors. Microbiologists have not contrived a unique terminology for these behaviors simply because the behaviors are exhibited by prokaryotes rather than metazoans. Similarly, there is no a priori reason to apply a different terminology to bacterial interactions bearing the exact same hallmarks of envenomation seen in animals. Thus, bacteria that exhibit toxin delivery mechanisms fully analogous to those of animals should be considered venomous.

Predatory bacteria occur in at least 15 families of five phyla and occupy virtually every habitat, including rivers, groundwater, estuaries, open ocean, sewage, soils, plant roots, animal feces, and even within the mitochondria of *Ixodes* ticks [109]. It would seem surprising if these organisms did not evolve prey subjugation methods similar to those of their metazoan counterparts. Other forms of bacterial nutrition induce pathogenicity by the introduction of toxins, and venom delivery systems might exist in these groups as well.

**Figure 4:**
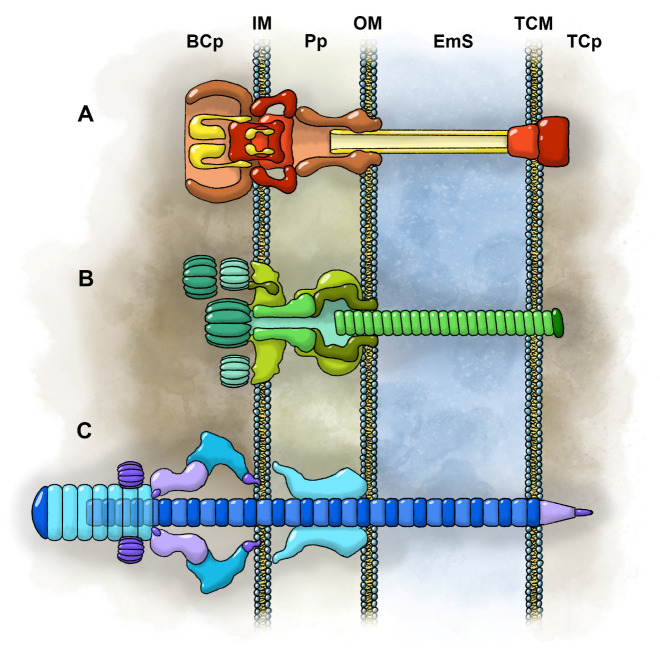
Three bacterial secretion systems that penetrate target cell membranes to inject toxins. (**A**) Type-III secretion system. (**B**) Type-IV secretion system. (**C**) Type-VI secretion system. BCp: bacterial cytoplasm. EmS: extramembranal space. IM: inner membrane. OM: outer membrane. Pp: periplasm. TCp: target cytoplasm. TCM: target cell membrane. Bacteria rely on these systems to introduce toxins into the cells of other organisms. Artwork: M. Benjamin Streit.

Bacteria cause numerous pathogenic conditions by secreting substrates, including “effector” proteins, that enter host cells and modulate cellular processes in their favor. As many as 10 secretion systems (including the chaperone/usher pathway) have been described from bacteria [110,111,112,113,114,115,116]. Of these, three involve sophisticated structures that ensure effector delivery into the cytoplasm of other cells: the type-III, type-IV, and type-VI secretion systems (see [117,118,119] for recent reviews). More than 350 effector proteins have been described [110]. Effector proteins can alter virtually every aspect of the host cell’s physiology, often resulting in devastating diseases of plants, animals, and even humans. Some effectors manipulate host cell actin polymerization mechanisms to promote uptake of the bacteria, which can then replicate and further propagate pathogenicity [117,120,121]. These sophisticated nanomachines constitute a venom delivery system if toxin delivery takes place via a “wound” in the target (Figure 4).

Here, we describe three categories of candidate venom delivery systems in bacteria: type-III secretory systems (T3SSs), type-IV secretory systems (T4SSs), and contractile injection systems (CISs). The latter group includes type-VI secretory systems (T6SSs). Bacteria that possess these systems can be considered autoaglandular–venomous.

### 6.1. Type-III Secretion Systems (T3SSs)

Type-III secretion systems have been described from more than 25 species of Gram-negative bacteria that interact with live nematodes, insects, plants, and animals. The T3SS apparatus, specifically the injectisome or needle complex, spans the bacterial inner and outer membranes and projects externally as a needle (Figure 4A). This device, which is structurally similar to the flagellum, allows the bacteria to dock with the cell surface of its target and provides a conduit or pathway by which toxin may be delivered to the cytosol or cytosol surface of the target [118,122,123,124]. Two substrate types are secreted: the aforementioned effectors and translocators, which are secreted ahead of the effectors to complete the needle tip and facilitate the delivery of effectors across the target’s envelope [125,126]. Despite previous concerns raised by the fact that effector protein toxins are too large to fit through the needle pathway, more recent work using cryo-electron microscopy has provided direct evidence that the effector proteins do pass through the needle pathway in an unfolded state [116,127,128,129].

### 6.2. Type-IV Secretion Systems (T4SSs)

Type-IV secretion systems occur in Gram-positive and Gram-negative bacteria and in a few archaea. The highly variable structure, which also spans the inner and outer membranes (Figure 4B), can be used to transfer DNA and diverse effector proteins into other bacterial or eukaryotic cells, but translocation does not always require contact with the target [119,130,131,132]. Contact with the target occurs through a conjugative pilus in Gram-negative systems or by surface adhesins in Gram-positive systems [131]. One of the best-studied systems involving the T4SS is the plant pathogen *Agrobacterium tumefaciens*. This bacterium uses the T4SS to inject a tumor-inducing (Ti) plasmid that causes a disease called crown gall. The plasmid not only induces tumor formation but also causes the plant to produce amino acids and sugar–phosphate derivatives (opines) that the bacteria can use as a food source [133]. Given the clear pathophysiological effects of the Ti plasmid on a host plant, this provides a case where DNA might be considered a venom.

### 6.3. Contractile Injection Systems (CISs)

Contractile injection systems comprise a larger class of nanomachines that are involved in the injection of toxins into other cells. They share structural components similar to the contractile tails of phages [117,134,135]. They consist of an inner tube surrounded by a contractile sheath, a spike capping the inner tube, and a baseplate complex at the base of the sheath. Contraction of the sheath propels the inner tube into the target, delivering the effector protein toxins. The CISs are divided into two main categories based on their mode of action: extracellular CISs (eCISs) and type-VI secretion systems (T6SSs) [134,135,136,137].

eCISs have been characterized as “headless phages” that are released by bacteria into the surrounding medium, bind to a target cell’s surface, and then inject their payload [134]. Diverse eCIS structures have been described, including contractile R-tailocins, antifeeding prophages (Afps) of *Serratia entomophila*, *Photorhabdus* virulence cassettes (PVCs), and metamorphosis-associated contractile structures (MACs) of *Pseudoalteromonas luteoviolacea* [135]. Although several roles have been proposed, their release may represent a self-sacrificial mechanism by a small proportion of the population to protect and benefit the entire population from competitors [135,136]. Genome-based analysis has revealed that the genes for such structures are widely distributed among various groups of Gram-positive and Gram-negative bacteria, as well as certain groups of archaea [138].

Type-VI secretion systems (T6SSs) are defined by their intracellular localization and attachment to the cytoplasmic membrane [134]. As membrane-penetrating nanomachines (Figure 4C), they are employed by Gram-negative bacteria to target a wide range of both prokaryotic and eukaryotic cells. By some estimates, this recently discovered and highly versatile envelope-spanning structure accounts for up to a quarter of all Gram-negative secretion systems [117,139,140].

The T6SS pathway appears to rely on direct contact with the target cell’s surface. According to the current model [117,141,142], the external portion of the delivery complex anchors to the target cell’s membrane. Once anchored, it inserts a membrane-spanning complex and then contracts to eject two associated effector proteins—hemolysin coregulated protein (Hcp) and valine–glycine repeat protein G (VgrG)—in a single step through both the inner and outer cell membranes of the target. Subsequently, the delivery complex disassembles at least partially for a new round of firing. Some pathogenic bacteria also deliver classic toxins along with Hcp and VgrG [141,143]. Accumulating evidence suggests that T6SS serves primarily as a device for inter-bacterial competition [117]. In one remarkable example, *Pseudomonas aeruginosa* unleashes lethal T6SS retribution against heterospecific bacteria that attack them first, while ignoring other harmless, potentially helpful neighboring bacteria [144,145].

## 7. Viruses

**Figure 5:**
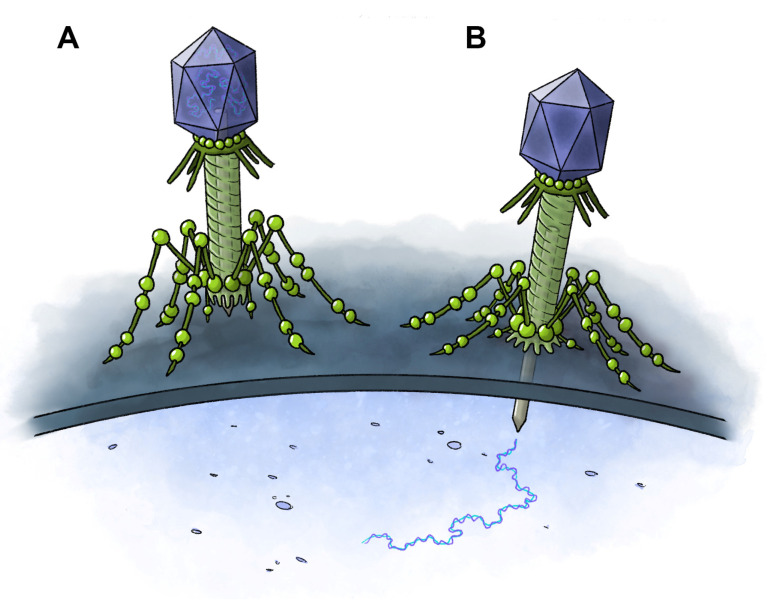
Proposed venom delivery system of bacteriophage viruses. (**A**) Bacteriophage prior to injection of DNA into target cell. (**B**) Bacteriophage following injection of DNA. The DNA causes damage to the host which rivals that of many conventional venoms. Artwork: M. Benjamin Streit.

The term “virus” entered English in the late 14th century, meaning “venomous substance,” derived from the Latin word *virus*, meaning “poison, poisonous liquid, sap of plants, slimy liquid, a potent juice” [146]. By about 2500 BC, the Chinese had identified smallpox and recognized that it was transmissible, and Aristotle (384–322 BC) was aware that rabies was transmitted by dogs [147]. Not until 1728, however, was virus used in its more contemporary meaning: an agent that causes infectious disease [146]. Despite its etymology and history, the term virus is not normally associated with the term venom. Nevertheless, at least one good candidate for a venom delivery system exists within this group (Figure 5).

Many argue that viruses are not true organisms since they lack cellular structure and are incapable of independent metabolism. As “organisms at the edge of life” [148], they require the machinery of a host cell to make new products and to reproduce. They accomplish this by invading the cell and releasing nucleic acids that manipulate their host. Viruses invade host cells by numerous means, which usually involves penetrating the host membrane via fusion, pore formation, or endocytosis [149,150]. Plant viruses, unable to penetrate the cell walls of their hosts, often manipulate insects to inject them into the cells through their proboscis or other mouth parts [151]. These mechanisms do not resemble venom delivery.

However, one mechanism of cell entry, exhibited primarily by DNA bacteriophages (which infect bacteria), requires the virus to attach itself to the host cell’s exterior and inject its DNA genome along with some virion proteins through the host envelope into the cytoplasm using a hypodermic needle-like apparatus [152,153,154,155,156,157]. This process involves, at least in part, high pressure within the viral capsid to help propel the genome through the host cell’s envelope, but other forces may be necessary to complete the process. This form of wound creation and delivery by injection provides a clear analogy to the venom delivery systems of animals.

Although nucleic acids are not normally considered a toxin, the damage inflicted to the host cell, largely via products encoded by the bacteriophage genome, rivals that of many conventional venoms. The injected genome immediately arrests host gene expression. It then initiates its own enzyme synthesis, often producing toxins [158,159], and it can begin the replication of its own DNA and the formation of new virus particles, culminating with lytic rupture of the host cell to release 100–150 progeny viral particles within 30 min or less [160]. The notion of viruses functioning as toxins was proposed long ago [161].

Viruses occupy virtually every ecosystem on earth, with bacteriophages—at an estimated 10^31^ particles (roughly a trillion for every grain of sand)—comprising the most abundant biological entity on our planet [162,163,164,165]. By the consensus criteria for a venom delivery system, viruses could be regarded as the most abundant venomous life form on this planet.

## 8. Conclusions

Identifying venom delivery systems and venomous entities depends on a generally accepted definition of venom. The definition we have applied—a toxic secretion conveyed to internal tissues via the creation of a wound—clearly represents the consensus position [10], though some [11,12,13,14,15,16] have argued that the definition must also require an identifiable function, or adaptive role, which is seldom demonstrated and can be difficult to achieve. The requirement implicitly creates a fourth, presumably very rare toxin class: a secretion that is injected but has incidental toxicity. The requirement also raises the question of whether a functional role should be required for the definitions of poison and toxungen, as well as how many classes of biological toxins are desirable. Moreover, Gödel’s Incompleteness Theorems [166] suggest that no mathematical system can explain all observations, as there will always be statements that are not provable from within the system. If we extend this theorem to biology, which we feel is warranted [167], we may never be able to agree on a definition that addresses all observations. Even so, many of the candidate venom delivery systems we have proposed in non-animals provide an obvious functional role in defense, predation, competition, and/or reproduction. When assessing candidate venoms and venomous organisms, an effort should be undertaken to assess the presence of a delivery system, toxicity in the targeted system (whether the secretion causes pathophysiological effects), and, if feasible, a functional role for the secretion.

In our opinion, the evidence is overwhelming that numerous plants, fungi, protists, bacteria, and even viruses (though generally disregarded as living organisms) possess toxin delivery systems that are fully analogous to and often rival the complexity of venom delivery systems in animals. No rational reason exists to exclude these entities from dialogue regarding the evolution and nature of venoms, venom delivery systems, and venomous organisms. With venom considered a remarkable adaptation with numerous independent origins, our current appreciation of it has been based on a mere fraction of the evolutionary events that have culminated in its usage. Many of the evolutionary processes that have led to venom reliance will likely prove similar among the different taxonomic groups.

This newly recognized diversity of venomous organisms will take some time to become acknowledged and adopted. Those who study non-animal life forms will be slow to learn that some or many of the organisms they study should be deemed venomous, and a campaign should be undertaken to educate these specialists. The compartmentalization of researchers investigating different taxonomic groups has no doubt influenced the lack of convergence on similar terminology.

Eventually, specialists who study non-animals will contribute new knowledge and reshape the content of meetings and publications that focus on toxinology. We can expect to learn many new and exciting things about the evolutionary pathways of venom divergence, including gene duplication, the co-option of existing genes, and natural selection; the coevolution of venom and venom resistance; the energetics of venom production, storage, and replenishment; the ecological benefits and costs of venom use, including symbioses, aposematism, and mimicry; the behavioral aspects of strategic venom deployment, including venom metering, social hunting and defense, and roles as semiochemicals; and potential applications for biotechnology and human therapeutics. The volume of literature among the different groups we have explored is vast, so we feel like we have just scratched the surface with our effort.

Given the remarkable diversity of venomous organisms, we should not be surprised that toxungenous organisms also exist among non-animals. We urge investigators to explore this diversity as well. To inspire the effort and further clarify the distinction between venoms and toxungens, especially in unicellular organisms, we briefly describe here a few examples. For plants, toxungen use may be widespread among the roughly 21,500 species belonging to 40 families that secrete latex [168]. The secretion is stored under pressure in laticifer cells and, upon release following mechanical damage, often exerts antifungal and insecticidal properties that provide an effective defense against herbivores and pathogens [169]. A second example involves the parasitic mistletoe *Viscum album*, which is both toxungenous and venomous. The parasite produces xyloglucan endotransglycosylases and other effectors that degrade the cell walls of the host plant during the early stages of haustorium penetration (hence, a toxungen), and additional toxins can then enter the target through the haustorium-generated wound (hence, a venom) [170]. Among fungi, both entemopathogenic [79,80,81,82,83,84,85] and nematopathogenic [171,172] fungi can release toxins and/or hydrolytic enzymes to degrade the exterior of their targeted prey prior to hyphae invasion. More than 100 yeast killer species (non-hyphal fungi) similarly secrete killer toxins in targeted attacks of other microbes [173]. Among protists, a number of microalgae, including the dinoflagellate genus *Karlodinium* mentioned earlier, secrete extracellular toxins (exotoxins) into the surrounding water for prey immobilization and capture, predator deterrence, and competitor suppression [102,103,174,175]. Bacteria have evolved an impressive diversity of specialized toxungen delivery systems. Predatory bacteria can lyse the membranes of target cells to deliver toxic effectors via extracellular membrane organelles (outer membrane vesicles (OMVs) and membrane vesicles (MVs)) [176,177] and by epibiotic attachment, periplasmic invasion, or cytoplasmic invasion [105,178,179]. Cannibalistic bacteria likewise damage target cell membranes via toxins secreted by ATP-binding cassette (ABC) transporters [180]. Similar to protist microalgae, cyanobacteria secrete exotoxins (cyanotoxins) into the water that function in defense and competition [175]. Collectively, these examples support our view that diverse organisms have independently invented not just venoms but toxungens as well.

Clearly, animals share much in common with other life forms regarding their reliance on toxic secretions to solve critical problems such as predation, defense, and competition. The words of the Sherman Brothers in Disney’s iconic tune, *It’s a Small World*, could hardly be more apt: “There’s so much that we share, that it’s time we’re aware, it’s a small world after all”.

## Data Availability

No new data were created or analyzed in this study.
